# Multi-infection screening for migrant patients in UK primary care: Challenges and opportunities

**DOI:** 10.1016/j.jmh.2023.100203

**Published:** 2023-11-04

**Authors:** Jessica Carter, Felicity Knights, Anna Deal, Alison F Crawshaw, Sally E Hayward, Rebecca Hall, Philippa Matthews, Farah Seedat, Yusuf Ciftci, Dominik Zenner, Fatima Wurie, Ines Campos-Matos, Azeem Majeed, Ana Requena-Mendez, Sally Hargreaves

**Affiliations:** a The Migrant Health Research Group, St George's, University of London, London, United Kingdom; b Faculty of Public Health and Policy, London School of Hygiene and Tropical Medicine, United Kingdom; c Islington GP Federation, United Kingdom; d Refugee Council, United Kingdom; e Global Public Health Unit, Wolfson Institute of Population Health, United Kingdom; f Department of Health and Social Care, Office for Health Improvement and Disparities, United Kingdom; g Department of Health and Social Care, Addictions and Inclusion Directorate, Office for Health Improvement and Disparities, United Kingdom; h Department of Primary Care and Public Health, Imperial College London, United Kingdom; i Barcelona Institute for Global Health (ISGlobal Campus Clinic), Spain; j Department of Medicine Solna, Karolinska Institutet, Stockholm, Sweden

**Keywords:** Infectious disease screening, Migrant, Primary care, Digital tools, Health equity, Tuberculosis, HIV, Hepatitis B, Hepatitis C, Parasitic infection

## Abstract

**Background:**

Migrants in Europe face a disproportionate burden of undiagnosed infection, including tuberculosis, blood-borne viruses, and parasitic infections and many belong to an under-immunised group. The European Centre for Disease Control (ECDC) has called for innovative strategies to deliver integrated multi-disease screening to migrants within primary care, yet this is poorly implemented in the UK. We did an in-depth qualitative study to understand current practice, barriers and solutions to infectious disease screening in primary care, and to seek feedback on a collaboratively developed digitalised integrated clinical decision-making tool called Health Catch UP!, which supports multi-infection screening for migrant patients.

**Methods:**

Two-phase qualitative study of UK primary healthcare professionals, in-depth semi-structured telephone-interviews were conducted. In Phase A, we conducted interviews with clinical staff (general practitioners (GPs), nurses, health-care-assistants (HCAs)); these informed data collection and analysis for phase B (administrative staff). Data were analysed iteratively, using thematic analysis.

**Results:**

In phase A, 48 clinicians were recruited (25 GPs, 15 nurses, seven HCAs, one pharmacist) and 16 administrative staff (11 Practice-Managers, five receptionists) in phase B. Respondents were positive about primary care's ability to effectively deliver infectious disease screening. However, we found current infectious disease screening lacks a standardised approach and many practices have no system for screening meaning migrant patients are not always receiving evidence-based care (i.e., NICE/ECDC/UKHSA screening guidelines). Barriers to screening were reported at patient, staff, and system-levels. Respondents reported poor implementation of existing screening initiatives (e.g., regional latent TB screening) citing overly complex pathways that required extensive administrative/clinical time and lacked financial/expert support. Solutions included patient/staff infectious disease champions, targeted training and specialist support, simplified care pathways for screening and management of positive results, and financial incentivisation. Participants responded positively to Health Catch-UP!, stating it would systematically integrate data and support clinical decision-making, increase knowledge, reduce missed screening opportunities, and normalisation of primary care-based infectious disease screening for migrants.

**Conclusions:**

Our results suggest that implementation of infectious disease screening in migrant populations is not comprehensively done in UK primary care. Primary health care professionals support the concept of innovative digital tools like Health Catch-UP! and that they could significantly improve disease detection and effective implementation of screening guidance but that they require robust testing and resourcing.

## Background

Migrant populations residing in Europe are disproportionately affected by infectious diseases (ID) such as tuberculosis, HIV, parasitic infections and hepatitis B and C (Prevention ECfD, Control 2018; Noori et al., 2021; Aldridge et al., 2018; Greenaway and Castelli, 2019). The European Centre for Disease Control's (ECDC) latest guidance (panel 1, 2018) on ID screening and vaccination in newly arrived migrants stresses the need for holistic and innovative approaches to the provision of multi-disease screening and preventative healthcare (Prevention ECfD, Control 2018). This is reflected in national guidance from the UK Health Security Agency (UKHSA) migrant health guide (Office for Health Improvement and Disparities, Migrant health guide, 2022) and is in line with the WHO Global Health Sector Strategy on infectious diseases which aims to eliminate tuberculosis, HIV, and viral hepatitis B and C as public health problems by 2030 which includes a target to reduce these infections diseases among persons, including migrants' population everywhere (WHO, 2015, WHO, 2016). Despite the ECDC's and UKHSA's guidance, ID screening coverage in at-risk migrant populations in many high-income countries remains low (Seedat et al., 2018). A review of ID interventions for migrants across the EU highlights the challenges of disparities in treatment rates of diseases between and within migrant populations, and the need for implementation strategies that address migrant and practitioner knowledge, fear, and access barriers to health services (Driedger et al., 2018). The COVID-19 pandemic has further highlighted the barriers to health institutions and preventative healthcare that these groups often face on arrival to European countries, including physical barriers to services but also a lack of awareness amongst front-line healthcare providers and administrative staff of the health and access needs of this group, and lack of innovation in service delivery models to ensure they are included (Hayward et al., 2021).

**Table:** 

**Panel 1: ECDC Evidence-based statements regarding provision of infectious disease screening and catch-up vaccination for migrants, reproduced with permission]** (Prevention ECfD, Control 2018). Active TB Offer active TB screening using chest X-ray (CXR) soon after arrival for migrant populations from high-TB-incidence countries. Those with an abnormal CXR should be referred for assessment of active TB and have a sputum culture for Mycobacterium tuberculosis Latent TB infection Offer LTBI screening using a tuberculin skin test (TST) or an interferon-gamma release assay (IGRA) soon after arrival for all migrant populations from high-TB-incidence countries and link to care and treatment where indicated. HIV Offer HIV screening to migrants who have lived in communities with high HIV prevalence (≥1%). If HIV positive, link to care and treatment as per clinical guidelines. Offer testing for HIV to all adolescents and adult migrants at high risk for exposure to HIV. If HIV positive, link to care and treatment as per clinical guidelines. Schistosomiasis Offer serological screening and treatment (for those found to be positive) to all migrants from countries of high endemicity in sub-Saharan Africa, and focal areas of transmission in Asia, South America and North Africa. Strongyloidiasis Offer serological screening and treatment (for those found to be positive) for strongyloidiasis to all migrants from countries of high endemicity in Asia, Africa, the Middle East, Oceania and Latin America. Hepatitis B Offer screening and treatment for hepatitis B (HBsAg and anti-HBc, anti-HBs) to migrants from intermediate (≥2%) or high (≥5%) HBsAg prevalence countries. Offer hepatitis B vaccination series to all migrant children and adolescents from intermediate (≥2%) or high (≥5%) HBsAg prevalence countries who do not have evidence of vaccination or immunity. Hepatitis C Offer hepatitis C screening to detect HCV antibodies to migrant populations from HCV-endemic countries (≥2%) and subsequent RNA testing to those found to have antibodies. Those found to be HCV RNA positive should be linked to care and treatment. Vaccine-preventable diseases. Offer vaccination against measles/mumps/rubella (MMR) to all migrant children and adolescents without immunisation records as a priority. Offer vaccination to all adult migrants without immunisation records with either one dose of MMR or in accordance with the MMR immunisation schedule of the host country. Offer vaccination against diphtheria, tetanus, pertussis, polio and Haemophilus influenzae type b/HiB (DTaP-IPV-Hib) to all migrant children and adolescents without immunisation records as a priority. Vaccination against Hib is only recommended for children up to five years of age. Offer vaccination to all adult migrants without immunisation records in accordance with the immunisation schedule of the host country. If this is not possible, adult migrants should be given a primary series of diphtheria, tetanus, and polio vaccines. For hepatitis B vaccination, please see evidence-based statement for hepatitis B.

In the UK, initiatives to improve ID screening in primary care have included an increased emphasis on screening for latent TB, (Hayward et al., 2021) developing clear national guidance for clinicians, (Seedat et al., 2014) and the recommendation of an ‘extended’ New Patient Health Check for migrants registering in GP practices to explore broader health needs and tackle barriers to health care this population typically experience (Eborall et al., 2020). However, IDs screening and management often continue to be perceived as the realm of secondary care, addressed in single disease programmes, or missed entirely and studies demonstrate that there are significant health inequalities in screening provision (Hargreaves et al., 2014; Bil et al., 2018; Office for Health Improvement and Disparities, Migrant health guide, 2022; Hargreaves et al., 2020; Zuure et al., 2013).

Combining screening for multiple key infections in migrant groups in a primary care-based setting could prove an effective strategy. Initial studies suggest better uptake, feasibility and acceptability in integrated approaches (Seedat et al., 2014; Eborall et al., 2020; Hargreaves et al., 2014; Zuure et al., 2013). A pilot clustered-RCT of an algorithmic digital multi-disease screening tool amongst eight primary care centres in Catalonia, Spain showed increased screening and diagnostic rates for each infection included in the tool during the 9-month study period compared with a 6-years before the intervention implementation and demonstrated feasibility and acceptability amongst health professionals (Sequeira-Aymar et al., 2021; Greenaway and Hargreaves, 2022).

**Fig. 1:**
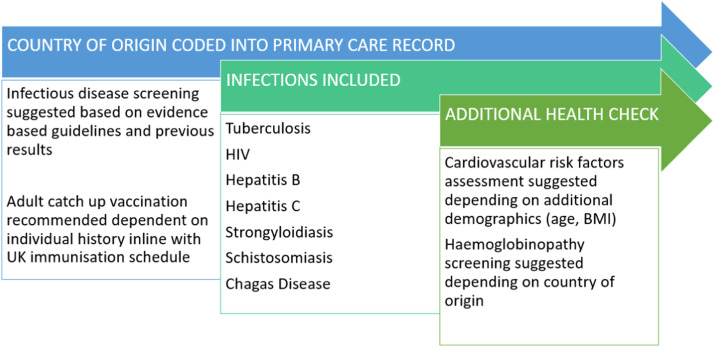
Health Catch UP! screening and catch-up vaccination prompts. Health Catch UP! Demonstration link: https://emishealth.vids.io/videos/a49ad1bb1a18e4c72c/health-catch-up-with-requested-edits-mp4.

A digitalised novel integrated screening tool, Health Catch-UP! for the UK has been developed prior to this study in collaboration with primary care teams, migrant patient representatives, academics, infectious disease experts, and Public Health England (the predecessor organisation to the UK Health Security Agency (UKHSA)). Health Catch-UP! facilitates systematic multi-disease screening for key infections alongside non-communicable diseases within the primary care setting. The tool aligns with national guidance on ID screening and catch-up vaccination and uses an algorithm based on country of origin that gives tailored and targeted prompts to the clinician on which IDs to screen for. It also prompts assessment of cardiovascular risk and haemoglobinopathy screening and indicates which adult catch-up vaccinations are required based on five key demographic variables (age, BMI, country of origin, ethnicity, and date of entry to the UK (which must be 5 years or fewer for LTBI screening)). Fig. 1 below summarises the key elements of the Health Catch UP! tool. This tool is integrated in EMIS (Egton Medical Information Systems), the most frequently used electronic primary care record system in the UK. Data are integrated with the existing clinical record and patient status regarding each condition/vaccination to ensure accuracy and non-repetition of prompts.

In this qualitative study we aimed to better understand primary care professionals experience and perceived role in infectious disease screening for migrant populations and address the gap in evidence for potential solutions to screening. We explored and assessed participants perceptions of current practice with respect to infectious disease screening and sought views on barriers, and facilitators to infectious disease screening for migrant patients in UK primary care. We also aimed to define targeted solutions to improving preventative health care and health outcomes for infectious diseases in these at-risk populations. Finally, we sought specific views on the infectious disease screening element of the Health Catch-Up! to inform development and implementation strategies for Health Catch-UP! to support UK primary care providers to better meet the needs of migrant patients. The vaccination component has been published in another paper (Carter et al., 2022).

## Methods

### Study approach

We followed the consolidated criteria for reporting qualitative research (COREQ) to design and report this study (Tong et al., 2007). We did a two-phase qualitative thematic analysis study using semi-structured interviews supported by field notes, using an iteratively developed topic guide (Table 1). In Phase A we interviewed clinical staff of GP practices which informed the topic guide for interviews in Phase B, where we interviewed administrative staff. The topic guides were piloted prior to data collection and enhanced through rephrasing and clarification, addition of further prompts and probes, and additional lines of enquiry throughout Phase A and Phase B.

**Table 1:** Topic guide subject matter for phase A and phase B.

Subject Matter	Included Questions	Phase A, Phase B or Both
Exploring personal and GP practice experience and knowledge of caring for migrant patients	*What proportion of your patients are migrants (born in another country)?* *What training have you had in relation to migrant health?* *Do you have any experiences you would like to share regarding providing care for migrant patients where this has gone particularly well? Or not gone well?* *Does your practice ask and then code country of origin?*	Both Phase A Phase A Phase B
Knowledge and experience of current practice of infectious disease screening for migrants	*Does any infectious disease screening take place at your practice currently?* *Are you aware of any guidance or interventions regarding infectious disease screening in migrants [at your practice or elsewhere?]*	Both Phase A
Explanation of the Health Catch-UP Tool	*We are generating a GP software based integrated health catch-up tool which incorporates vaccination catch-up and infectious disease screening based on country of origin It acts in a similar way to the over 75 health check (routine UK health check including* e.g. *cardiovascular risk) for example and it will prompt GPs and nurses if a patient is under immunised, or eligible for screening for key infections. It is highly targeted and generates information in one pop up which will summarise the blood tests, vaccines, and referrals needed.*	Both
Questions relating to Health Catch UP!	*What is your initial response to this description [of the Health Catch Up! intervention]?* *What would be some of the barriers and facilitators to a migrant health check using this tool?* *How do you think practice managers/ receptionists (relating to their job role) could support this type of health check?*	Both Both Phase B

### Research team

We developed a multidisciplinary research group to design and carry out this study. This comprised GPs (JC, FK, AM), health researchers with experience in migrant health and qualitative methodology (AD, AFC, SH), individuals with lived experience of migration (YC, MH), and experts in infectious and imported disease (ARM). The proposed study protocol was also presented and discussed at the St George's University of London Patient, Public Involvement and Engagement Board (SGUL PPIE) board to gain insights of individuals with a range of lived experiences of migration. The diverse backgrounds of the research team enabled detailed discussion and refinement of both the study methodology and topic guide.

### Study setting and participants

We recruited staff from 50 purposively sampled GP practices serving diverse populations, varied practice population sizes and from a range of urban, suburban, and rural settings across England, and interviewed staff from across the primary care team. Two practices were based in Oxford and Newcastle, the others were located within six local Clinical Research Networks (CRNs)—CRN Kent, Surrey and Sussex; CRN South London; CRN North Thames; CRN Northwest London; CRN West Midlands; and CRN Greater Manchester.

We carried out a total of 64 in-depth semi-structured interviews. Participants were recruited through CRNs, social media and primary care newsletters and through the process of snowballing sampling.

### Ethics and informed consent

Before data collection ethical approval was granted by St George's, University of London Research Ethics Committee (2020.00630) and the Health Research Authority (REC 20/HRA/1674). All individuals who expressed interest in participating were invited to arrange a telephone interview lasting up to an hour at a mutually convenient time. Participant information sheets were provided in advance and reviewed with the participant before the interview with opportunity to ask questions provided and written consent taken by the researcher conducting the interview (JC, FK, AD, AFC). Each participant was offered £20 voucher as compensation for their time.

### Data collection and analysis

At the start of the interview, we collected participant demographic information for each participant including age, gender, ethnicity, job title (general practitioners GP, practice nurses PN, health care assistants HCA (allied primary health care professional who supports work of the practice), administrative staff including practice managers and practice based pharmacists), size of practice (number of patients), rural/urban/suburb location, years in general practice and self-reported previous experience working in migrant health captured as free text.

JC and FK carried out most interviews with clinical staff, with AD and AFC supporting and interviewing a number of non-clinicians. The involvement of clinicians as interviewers has been thought to enhance qualitative health research through opportunities to build trust with clinicians, enhancing participation and exploration of sensitive issues, and by depth of understanding to the meanings practitioners bring to the health care environment (Greenhalgh and Taylor, 1997; Richards and Emslie, 2000). However, a reflexive approach by JC and FK who were cognisant of their own personal and professional experiences may influence data collection enabled improved probing, fewer assumptions, avoidance of premature interpretation; particular risks for clinical interviewers (McNair et al., 2008)The interviews were audio recorded with consent and then transcribed verbatim, checked for accuracy, and de-identified.

Data collection and analysis took place concurrently as is common in qualitative research, and data were analysed using thematic analysis, following the six steps set out by Braun and Clarke (2006). Data was managed throughout through NVivo 12 (QSR International, address). Collection ended when data saturation was reached, defined as the point at which additional data showed informational redundancy and further interviews ceased to generate new data as set out by Sandelowski (2008). We took a positivist approach to analysis, focusing on data which related to the broad aims of the study (primary care views on; current approaches to infectious disease screening for migrant patients, barriers and solutions to screening in primary care and responses to Health Catch UP! tool). JC undertook immersion, meticulously and systematically reading and re-reading the transcripts to gain familiarity. She then developed a list of initial semantic codes through an open coding approach, which were discussed with SH and FK. In addition, FK randomly chose 7 transcripts and applied JC's codebook, discussed the coding approach and discrepancies with JC, and this led to the amending of 1 code and addition of 2 codes with subsequent development of an agreed codebook. JC then identified emergent patterns, categories, and concepts to move beyond describing participant's comments to interpreting and explaining them. Themes were reviewed and refined by JC and FK through a series of discussions to generate a negotiated consensus, and following discussion with the wider research team, a final set of themes were defined and reported.

## Results

We did sixty-four interviews with staff from 50 practices across England. Phase A included 48 clinical primary care professionals (25 GPs, 15 practice nurses (PN), 7 HCAs and 1 clinical in-practice pharmacist). Phase B comprised 16 administrative staff (11 practice managers and 5 receptionists/other admin staff). Demographic data are summarised in Table 2. Most participants were female (84.4%) and had worked in primary care for over 10 years (mean 12.5, SD 9.64), 40% of participants self-reported experience in the field of migrant health (including volunteering e.g. doctors of the world, work abroad in refugee camp settings, academic global health research and working in inclusion health practices) and over 75% practiced in an urban setting.

**Table 2:** Participant demographic data.

	ALL	Phase A			Phase B
		GP	Health Care Assistant/ Pharmacist	Practice Nurse	Practice Manager/ Admin Team
**Number of participants n (%)**	64	25 (39.1%)	8 (12.5%)	15 (23.4%)	16 (25.0%)
**Age (mean, SD))**	45 (11.8)	44 (11.0)	41 (12.7)	45 (11.9)	48 (11.6)
**Sex n (%)** **Female** **Male**	54 (84.4%) 10 (15.6%)	17 (26.6%) 8 (12.5)	8 (12.5%) 0	15 (23.4%) 0	14 (21.9%) 2 (3.1%)
**Ethnicity n (%)** **African** **Any other Asian background** **Any other mixed background** **Any other white background** **Caribbean** **Indian** **Pakistani** **White British** **White Irish**	4 (6.2%) 2 (3.1%) 3 (4.7%) 5 (7.8%) 1 (1.6%) 11 (17.2%) 3 (4.7%) 32 (50.0%) 3 (4.7%)	0 1 (1.6%) 1 (1.6%) 1 (1.6%) 0 8 (12.5%) 2 (3.1%) 12 (18.8%) 0	1 (1.6%) 0 1 (1.6%) 1 (1.6%) 1 (1.6%) 0 0 2 (3.1%) 2 (3.1%)	3 (4.7%) 1 (1.6%) 0 2 (3.1%) 0 0 0 9 (14.1%) 0	0 0 1 (1.6%) 1 (1.6%) 0 3 (4.7%) 1 (1.6%) 9 (14.1%) 1 (1.6%)
**Years in general practice (mean, SD)**	12.56 (9.64 *n* = 62)	13.28 (9.78, *n* = 25	10 (7.62, *n* = 8)	11.38 (9.77) *n* = 13	13.66 (9.87 *n* = 16)
**Migrant health experience n (%)**	26 (40.62%)	13 (52%)	5 (62.5%)	7 (46.67%)	1 (6.25%)
**Number of Patients Registered at GP Practice** **<5000** **5000–10,000** **10,000–15,000** **15,000–20,000** **>20,000**	6 (9.4%) 24 (37.5%) 10 (15.6%) 15 (23.4%) 9 (14.1%)	1 (1.6%) 10 (15.6%) 6 (9.4%) 6 (9.4%) 2 (3.1%)	2 (3.1%) 3 (4.7%) 2 (3.1%) 1 (1.6%) 0	1 (1.6%) 2 (3.1%) 2 (3.1%) 4 (6.2%) 6 (9.4%)	2 (3.1%) 9 (14.1%) 0 4 (6.2%) 1 (1.6%)
**Location** **Rural** **Suburb** **Urban**	1 (1.6%) 13 (20.3%) 50 (78.1%)	1 (1.6%) 7 (10.9%) 17 (26.6%)	0 2 (3.1%) 6 (9.4%)	0 2 (3.1%) 13 (20.3%)	0 2 (3.1%) 14 (21.9%)

Three themes were generated from our data about infectious disease screening in migrant groups and converged across job roles (GPs, PNs, HCA, reception, practice managers), these comprised:

➢ Theme 1: Current approaches to infectious disease screening in primary care are inconsistent and non-standardised.
➢ Theme 2: Multi-level barriers (patient, staff, and system level) exist to primary care-based infectious disease screening.
➢ Theme 3: Primary health care professionals’ attitudes are positive to primary care's potential to effectively deliver infectious disease screening to migrant patients through innovative solutions including Health Catch-up!.

Theme 1 **Current approaches to infectious disease screening in primary care are inconsistent and non-standardised.**

Primary health care professionals highlighted diverse current policies to ID screening in general and for migrant patients within their practices (Table 3), demonstrating that UK primary care lacks a standardised approach. Screening practices reported ranged from a ‘best practice’ multi-disease universal screening policy at patient registration offered to all migrants to limited opportunistic ‘at risk’ testing usually for individual infections at the discretion of clinical staff within an appointment. This difference varied between practices and diseases. The majority of infections were reported as tested for in some format in most practices, with a particular focus on sexually transmitted diseases and blood borne viruses. There was a notable exception of parasitic infections which were only tested for in practices with GPs with specific interests or experience. Most practices had either no system or their own bespoke local or practice-level system in place. This suggests that many at-risk patients, including migrant patients, could be excluded from the screening recommended in national guidelines.

**PN2:** “If they specifically came and said, I'm concerned about hepatitis B, or something like that we would test. No, we wouldn't test a group of people just because they were from certain ethnic groups or migrants”.
**PN13:** [on screening for at risk hepatitis groups] “Within general primary care …I haven't seen increased screening unless they're [GP practice] in a specific [geographical] area [with a screening initiative].

**Table 3:** Current approaches to infectious disease screening described in UK primary health care professionals (supporting data available in Appendix 1).

Disease	Range of current reported approaches to ID screening by practices
**HIV**	Practice based risk stratification (high risk areas)
	Universal screening at registration for new patients
	Symptomatic screening by GPs*
**Tuberculosis**	Latent TB screening pathway for migrants arriving in the past 5 years
	Symptomatic screening*
	Not done / migrant is referred to secondary care
**Hepatitis B and C**	Universally completed at registration
	Abnormal liver function tests pathway prompting screening*
	Specific practice-based computer prompts from high-risk countries
**Sexually transmitted infections**	Young people screened through computer prompts/ patient health checks
	Patient request at registration
	Symptomatic *
**Malaria**	Unwell returning traveller*
	Patient request
**Parasitic infections (and other neglected tropical diseases (NTDs) including Chagas Disease, Strongyloidiasis, Schistosomiasis, Helminth infections, Leprosy, Yaws etc.)**	Indication from blood test (including eosinophilia or anaemia) *
	Risk stratification
	Not done /migrant is referred to secondary care

*It is of note that participants incorrectly described these reasons to test for infectious disease as ‘screening’.

Participants also reported inconsistent understanding and application of recommended screening for migrant patients in day-to-day practice. Many clinicians incorrectly described symptomatic testing or testing unwell returning travellers for malaria as screening. Even when clear guidance or screening pathways were in place and participants knew about these, both clinical and administrative staff often reported inconsistencies within and between practices with no clear rationale as to which screening protocols were followed and why. Furthermore, some participants reported that even when screening or testing is offered to a patient, the results for this are not always followed up and it isn't always known whether patients completed the tests or not.

**GP23:** “So if someone presented with restricted breath and a cough, then absolutely that would be part of it…. Absolutely at that point they do get fed into TB screening if that's relevant and TB vaccination”.
**GP14:** “The only thing locally that's been put in place already is the TB screening. So, if anybody's registering or newly registered with the practice who's been out of the country for five years, then they'll get a blood test form at the point of registration. But we don't necessarily actually follow it up or chase it at all because the funding's so small that once we've done the blood test form and we've sent them a text message to remind them, if they still haven't gone for it, we just left it!”.

Theme 2 **Multi-level barriers (patient, staff, and system level) exist to primary care-based infectious disease screening.**

Participants discussed multiple barriers to providing multi-infection screening within primary care with many participants expanding on the resultant missed opportunities and failures of current pathways. Barriers were reported at a patient level, staff level, and system level that merit further consideration (Table 4). Specific barriers to existing single infectious disease screening pathways are reported in Table 5. Respondents reported feeling that migrant patients often did not understand or prioritise infection screening and that in some cases staff were concerned about appearing to discriminate by offering infection screening to some patients and not others. Clinical staff reported that they do not see most infectious disease screening as part of routine care, or migrants as a priority group, and felt that they lacked the knowledge, experience, and in some cases the interest, to offer this. At a systems level, concerns were raised over the practicality to access some infectious disease screening tests locally, the lack of consistent guidelines and pathways, and the fact that positive results are often not fed back to the practice, with many clinicians concluding that it's easier to refer the patients to secondary care as a result. Illustrative quotes are supplied in Table 4.

**GP1**: “The patient facing challenge would be, it has to be a very simple and easily understandable process. And also, if they're doing multiple of the same tests… You can get a significant lack of uptake. They need to be tied in, in a universal, one set of tests and blood tests, or urine, would generally be very effective.”
**GP 8:** “The hard-to-reach population [migrants]. People just don't appreciate the [needs] for that group, basically. I think that's what it really boils down to. So, it doesn't become a priority on the [GP] syllabus, which is already overflowing with lots of stuff, basically. So, it doesn't really feature that much. It's the conflict between demand and need in my mind, basically. [Primary care] deals with demand, but if we're actually talking about people that need the care, we're not getting to that. We still haven't got to that”.
**GP 16:** “the simplest thing is for them to nip across to Hospital for Tropical Diseases”.

**Table 4:** Patient, staff and system level barriers to infectious disease screening in primary care.

Level	Barrier	Illustrative Quote
**Patient level**	Patient understanding of screening	Admin 4: Yes. I don't think a lot of countries… sometimes you don't have preventative measures in place, so trying to explain that and why we do them can be quite difficult.
	Concerns about discrimination	Admin 13: [about LTBI screening] patients said they felt singled out. I think some of them, it wasn't an easy conversation of saying to someone, you know, we think you might be infectious. Can we test you? And so, it was a conversation that we generally then left.
	Not a patient priority	GP 8: Yes, it's definitely not at the top of the list. I think they've got far bigger problems actually.
**Staff level**	Not a practice priority	GP 6: We're far more likely to be following our guidelines for hypertension or kidney disease guidance. Mainly because there's more likely to be a QOF [payment] indicator. [That is] unfortunately the way a lot of general practices work.
	Time intensive/ workload concerns	HCA 3: Just workload, I think, and more the concern out of workload. If people feel like it's going to put more strain and more work on their job, then there is always a reluctancy.
	Lack of knowledge or experience	GP6: It's a question of should we be screening for parasitic infections, I don't know. Because I don't know what the risks and benefits are. I don't know how many tests you need to do to get a positive result and if that's worth it. If we're screening then I imagine, there's criteria for screening, right?
	Lack of interest/leadership	GP17: It depends which HCA they see, but one is very keen and the other isn't. I'm afraid it's often a bit like that.
**System level**	Challenges in identification of patients at risk	Admin 12: The first barrier you'd come across is, how would you identify these patients once they register and what read coding, we're putting in place that will allow you to bring those people up?
	Practical barriers to accessing tests in primary care	GP17: “The IGRAs [a blood test for TB] have to be in by a certain time…There's the purely administrative issue about getting the IGRA bloods at the lab at the right time.”
	Lack of prioritisation of migrant groups	GP 12: [Staff are] open to providing good care to this group but have to be realistic that prevention and vulnerable patients is not a priority…
	Not incentivised by primary care management systems /part of current routine care	GP2: I think you need some backing from the CCG [Clinical Commissioning Group]. Practices probably won't do this [extra work]. I think, really, ideally, if it's not part of an LCS [locally commissioned service], the hurdles are huge. If it's just something that's at the goodwill of GPs, I think you're going to struggle.
	No feedback on positive results	Admin13: I think another issue that the results wouldn't come back to the practice after they got sent off.
	Lack of consistent guidelines/ pathways and sustained initiatives	ADMIN 1: [with regards screening interventions] They run these campaigns and then suddenly it fizzles out and it doesn't come back again.
	Remit of secondary care	HCA 7: No, no. We send them to the hospital; we send them to the infectious diseases department. We used to do blood tests for TB for people who come from Africa, or any other part of the world with TB, but now we stopped. So, we just give them the number, and they go to the hospital.

**Table 5:** Key barriers described by UK primary health care professionals within specific existing infectious disease pathways.

Pathway	Reported Reasons for Screening Pathway Failure
LTBI	• Overly complex pathway. • Unable to identify patients at risk due to lack of coded country of origin. • Difficulty explaining TB risk to patients (non-clinical staff offering test, language barriers). • Stigma of patients being singled out for TB test • Lack of understanding of LTBI from clinicians and patients. • Overly restrictive criteria for testing (requiring knowledge of patient's age and number of years in the country: clinicians stated these were hard to ask and extra stages to deliver test) • Difficulty organising IGRA blood test (timing of couriers, availability phlebotomy, GP unable to order, local lab restrictions)
Universal HIV testing	• Lack of reception/HCA confidence offering HIV testing at registration. • Temporary funding for increased testing withdrawn
Abnormal liver function tests prompting hepatitis screening	• No feedback to local staff on the impact and next steps from positive results. • Temporary additional funding meaning practices bearing some costs if continuing the initiative • Overly complicated template to request tests. • Difficulty contacting patients in particular those testing positive due to a hard-to-reach group difficult to engage in treatment and follow up. • Pop up fatigue on the computer IT system requesting screening. • Perceived lack of secondary care support to receive patients testing positive
Chagas disease	• Electronic challenges in requesting the appropriate blood test on GP systems (Trypanosoma cruzi serology). • Lack of dedicated champions with high levels of knowledge regarding the condition and screening pathway
Sexually Transmitted Infections	• Pop up fatigue on the computer IT system requesting screening. • Reduced funding for specialist STI screening initiatives. • Lack of patient engagement in receiving screening for STIs by all patients

**Fig. 2:**
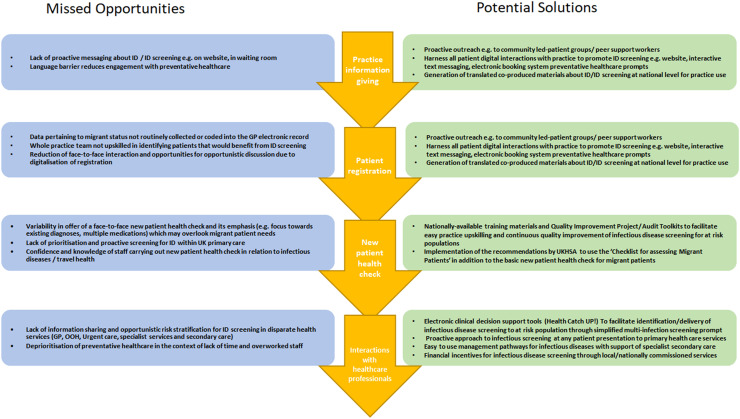
Missed opportunities and potential solutions for ID screening reported by primary health care professionals in patient pathway in primary care.

Missed opportunities for screening and potential solutions were highlighted throughout the patient pathway, these opportunities and solutions have been summarised in Fig. 2. A key moment for screening was felt by most participants to be at registration but that this often not done for multiple reasons. Administrative Primary Care Professionals (PCPs) working on reception citing lack of confidence in offering screening and concerns about patient privacy and stigma and all PCPs pointing out that much of the initial patient journey was migrating online with less face-to-face interactions available to offer screening.

Despite this many staff (clinical and non-clinical) reported willingness and eagerness to provide screening based on new initiatives including, inter alia, the push for universal HIV testing and the national LTBI screening programme. However, participants reported that these pathways were often overly complex, required large amounts of administrative and clinical time, and that financial and expert support was lacking. The LTBI pathway was most mentioned as poorly adhered to. In comparison, viral hepatitis testing (rather than screening) following prompts due to abnormal liver function tests was more successful in certain areas. The major pathway initiatives and the most common difficulties faced have been summarised in Table 5 below.

**ADMIN 6:** “[LTBI pathway] … But it never took off. It never really went anywhere, so even though sometimes people are making pathways, it was overly complex and couldn't be delivered by the practice.”
**ADMIN 13:** “[viral hepatitis pathway] …I think that it was too complicated, and the communications weren't very clear.”
**ADMIN6:** “[LTBI pathway] …Some of it was difficulties with the receptionist consistently doing that. So consistently getting out the flags of the world and then having to communicate to the patient why they were asking them to point at flags, what the purpose of that activity was, was quite a challenge.”

➢ **Theme 3: Primary health care professionals’ attitudes are positive to primary care's potential to effectively deliver infectious disease screening to migrant patients through innovative solutions including Health Catch-up!**

### Potential solutions and innovations

Participants overwhelmingly reported positive attitude towards infectious disease screening in migrant populations and a desire to change what many perceived as an inadequate current service. These included taking a pro-active approach to fostering greater patient understanding of holistic care that includes infectious disease screening from registration onwards, identification and practical support of patient or staff champions to increase the profile of infectious disease screening at population or practice level, and the use of targets or financial incentivisation at a higher level than the practice (e.g. Primary Care Network, Clinical Commissioning Group) to ensure ring-fencing of time and prioritisation of this previously neglected area of clinical provision until it is seen as ‘routine care’.

**GP1**: “[to be effective] They [migrant patients] have to understand the relevance of it [infectious disease screening] from the outset. Chasing them up later on is a real challenge. So, it's doing it at the start, and saying… We're doing this as a proactive health check, health screen, to try and look for you, and look for these infectious diseases… It's a golden opportunity, right at the beginning of their journey into the NHS system.”
**GP4:** “I think to be very honest with you, it all comes down to money. Because if you are a good, conscientious GP, you will do it much more without being asked. But you have to understand that you can't keep loading the camel with more and more straw. So, at some point it is going to break its back, however strong the camel is.”

There was almost universal consensus that the existence of an established staff practice champion supported normalisation and integration of infectious disease screening into day-to-day care and was essential in providing feedback on positive results and delivering next steps for care of patients testing positive. The loss of this champion often resulted in the drop-in rates of screening at the practice.

**PN7:** “Yes, we have a GP that is really passionate about this, [infectious disease screening] and she's always encouraging all of us and seeing it's made part of the [practice] policy, so it's great.”
**GP22:** “We did have an F2 [junior doctor in training] who was very keen on Chagas disease, so we did try and implement a lot of screening for that. But it's stopped since they've gone. He left recently. I think we just all forgot to do it.”

A wide range of facilitators/ solutions and innovations were described and suggested these have been summarised by patient, staff and system level in table 6. A full range of responses are reported in Appendix 2.

**Table 6:** Identified solutions and innovations to multi-disease screening in primary care *(Supporting data available in*Appendix 2*)*.

Level	Solution
Patient Level	Patient engagement using trusted sources from within the community patient-led groups and outreach
	Foster greater patient understanding of holistic multi-infection screening approach, and its benefits, at first engagement with the GP practice using co-produced translated materials
	Explore the role of using patient champions to support multi-infection screening and preventative healthcare in migrant patients
Staff Level	Identification and practical support of a dedicated member of staff at practice level to oversee training, delivery of services, liaising with secondary care, and updating of clinical pathways according to changing guidelines, leading liaison with secondary care
	Practice Quality Improvement Projects specifically aiming to improve understanding and practice around infectious disease screening
	Specific infectious disease clinics by GP with special interest in high-risk areas
	Feedback to team of positive results and the impact of these
	Engagement of whole primary care team and practice-wide protocol
System Level	Patient-facing campaigns to improve population understanding of infectious disease screening translated into multiple languages
	Specialist support such as upskilled primary care staff or secondary care outreach clinics
	Collaborations between several GP practices to focus on providing services for a larger population (such as through Primary Care Networks or Clinical Commissioning Groups)
	Use of targets or financial incentivisation at local or national level (i.e., a commissioned service)
	Easy screening pathways which provide all information or tests concurrently
	Easy access to tests and follow-up support/peer support for patients testing positive
	Computer prompts to support clinical decision making
	Competition between practices to demonstrate success of screening programmes

### The need for targeted, tailored training across the practice team

The majority of staff identified a knowledge gap in delivery of migrant infectious disease screening and felt that training opportunities was essential to ensure improvement in service delivery and reduce health inequity. This training should be integrated into curricula across the practice team and include an explanation of the benefits of infectious disease screening for the patients and the practice itself. Clinical staff particularly highlighted the need to improve awareness of infectious disease prevalence in their patient populations and risk stratification approaches. Administrative staff also reported training needs in relation to broader migrant health approaches in primary care citing the hostile environment, language, and a lack of awareness of different cultural beliefs as barriers at the front desk prior to any clinical interaction/screening.

GP2: [Migrant health training] made me aware how endemic some of these infections were and issues were and made me think about how when these people come to the country or we're only seeing them for the first time, it's just worth considering their wider, wider health.
GP18: We have intermittent training on different topics, and we had one recently on TB, and they really highlighted different rates from different countries, and we realised actually our Filipino group of people that we meet are probably the highest risk. I had no idea how high the rate of TB was in the Philippines.
ADMIN 2: I think it will be really useful if there is training around this because as I said, I don't think many people take into account any cultural differences. So, everyone is treated the same, but when you have a different religion, different cultural background, it would be useful to have some knowledge about in immigrant healthcare, actually. And as administrators, usually we are the first point of contact, then it would be… Especially, for example, if we have to call patients to do immunisations or we call patients to do [health] checks or things, if we have a training, it would be easier for us to get everyone involved.

### Specific responses to the potential of the health catch-up! tool

Participants’ responses to the description of the Health Catch-UP! Tool during interviews was overwhelmingly positive. In principle respondents felt that the tool represented good preventative patient centred health care for a vulnerable group who were often ignored or left out of such initiatives. They also felt that Health Catch-UP! would bypass a lot of the barriers identified to infectious disease screening and enable a systematic integration of information and prompts to support effective clinical decision-making for migrants.

**GP15:** “think it's quite easy to justify it being a pretty key part of the primary care we can give to migrants, isn't it? I think waiting for people to present with stuff, particularly passive case-finding … is a really bad idea on a public health basis, not just on an individual health basis.”
**PN 12:** “[Health Catch-UP!] is very much a one-stop shop for all the clinicians, and it's all on one page. So, anything that can help … generate all the information on one page, it would be great and I'm sure high in demand.”

Participants felt that by specifically identifying and systematically coding country of origin into notes and then highlighting those who may be at risk of certain infections, would raise awareness amongst staff and ease implementation of screening.

**PHARM 1:”** [Health catch up would] make us more knowledgeable … and would be easier to be able to say to them [migrant patients] this is what we are offering and this is why…it's not one-size-fits-all, and anything that you can do to tailor it to the patient, that would be absolutely fantastic…. It sounds like a tool like this would be great in terms of tailoring it to the patients depending on their country of origin. That would be great. It would save us a lot of time as well.”

Respondents felt that the way the tool worked would make it easy to adapt and adopt to their individual practices which would give them ownership of the tool.

**PN4:** “It might be a generic template the PCN's are using but individual GP surgeries change it a little bit so adopt it to their own. So, yes, so that (Health Catch-UP!) could be something that each surgery can also do, adopt the template”

There were concerns highlighted around the capacity of primary care to deliver this tool effectively and safely. This was particularly because infectious disease screening for migrants is not seen as routine care, there is a depleted workforce and a lack of available appointments especially without additional financial reimbursement.

**GP6:** “Yes [infectious disease screening is a priority], because although part of me feels that's terrible and we should be doing that [infectious disease screening in migrants] already. If you're working with a workforce that's already overly stretched, in order to guarantee the practice is able to allocate time and resources for someone to do something extra, you have to justify it.”
**ADMIN 5:** “There will be quite a few patients that will need these health checks. So that will definitely be a challenge.... Making sure everybody's trained, so they know exactly what these patients need.”

Respondents emphasised the need for adequate resourcing to implement the tool through training, financing and robust management pathways for any pathology found.

**ADMIN 15:** “It might be a costly and expensive project, but if there is funding available for the practices to target the key demographics or key populations, then, yes, I think there'll be pretty good uptake on it from the practices’ point of view.”

Participants key concerns and suggested benefits of using the Health Catch-UP! tool have been summarised in Table 7.

**Table 7:** UK Primary health care professionals views on new digital tool: health catch-UP! (supporting data available in Appendix 3).

Level	Benefits	Concerns
**Patient Level**	Provision of good preventative care	Stigma of targeted screening
	One stop shop for patient (improved uptake)	Risk of DNA
	Focus on vulnerable group	Fear of sharing data (country of origin)
**Staff level**	Everything in one place	Pop-up fatigue on the IT system used in primary care
	Reduce workload (reduces need to search for information)	Increase workload (This screening isn't currently occurring)
	Supports clinical decision making	Lack of confidence in infectious disease/migrant health
**System Level**	Systematic approach (ensuring patients receive all the care they are entitled to)	Incentivisation/financing needed as this isn't seen as routine work
	Standardisation of screening/vaccination	Increased use of appointments (This screening isn't currently occurring)
	Increase data – improved care/ equality but allowing appropriate coding of data	Difficulty identifying patients due to lack of coding of key data

## Discussion

### Summary of main findings

Our study aimed to explore the views of primary care professionals on current screening approaches for migrant patients, including barriers and facilitators as well as opportunities to improve them. We found that current infectious disease screening in UK primary care lacks a standardised approach. Many GP practices have no satisfactory system for screening in place, and in turn migrant patients are not receiving the care recommended in national evidence-based guidance (Prevention ECfD, Control 2018; Office for Health Improvement and Disparities, Migrant health guide, 2022; Seedat et al., 2018; NHS England Tuberculosis Programme, 2022). Where screening does take place, there are multiple approaches both within and between practices, often with an individual infectious disease focus and so neglecting all infections migrant patients may be at risk of. Screening is often based on individual healthcare professionals’ interest and offered in an ad hoc and opportunistic fashion. We identified major barriers as follows: at patient, staff, and system levels to the delivery of infectious disease screening. Key barriers included that infectious diseases were not considered a priority for patients or health care professionals, overly complex pathways for delivery of screening and management of positive results by healthcare professionals, difficulty in identifying those at risk due to lack of routine data collection, and coding of country of origin into GP records. Evidence from interviews suggest there could be capacity issues in primary care due to reported high workload and lack of financial incentives. Primary care professionals felt that these barriers contribute to poor engagement with existing screening programmes and current interventions.

Despite the identified barriers, primary health care professionals were overwhelmingly positive about their ability to effectively deliver infectious disease screening within primary care and keen to engage with innovative digital solutions such as the Health Catch-UP! tool. Key facilitators and solutions were highlighted to delivery of infection screening including recruitment and support of patient and staff infectious disease champions, improved awareness and engagement of patients and health care professionals through targeted campaigns, provision of training and support materials, adequate resourcing and financial incentivisation and simplified screening and management pathways for positive results with specialist input. The concept of the multi-disease screening approach via the Health Catch UP! was seen as strengthening the primary care response to migrant health and infectious diseases. Primary care professionals suggested that the tool should be embedded at registration / new patient health check, that training and resources (financial and materials, and staff) would need to be provided for both staff and patients, and that robust management pathways would be required for the follow up care to ensure a complete screening programme.

### Links to previous literature

Our study is in line with previous research that show current infectious disease screening for migrants in primary care is inadequate, often limited to single diseases, primarily latent tuberculosis, that existing national guidelines are poorly implemented and barriers exist at multiple levels (Evlampidou et al., 2016; Seedat et al., 2018; Moonen et al., 2023; Sweeney et al., 2015; Seedat et al., 2014). However our study has furthered this work by providing evidence that primary health care professionals identify that they are well placed to deliver infectious disease screening for migrants, are enthusiastic to do so and there are multiple primary care initiated potential solutions.

With respect to improving screening for migrants, based on the views of migrant health leaders, Seedat et al. (2014) concluded that primary care services need to incorporate community collaboration and move towards multi-infection screening. Our study adds to this body of evidence based on the views of primary care staff. Primary care is uniquely placed to strengthen community engagement and develop innovative solutions with and for the local population and our study shows that primary health care professionals are keen to innovate in this area through implementation of integrated digital tools and working alongside patient/peer champions. Patient champions/peer support can actively engage with marginalised groups such as migrants through shared storytelling and shared lived experience overcoming many of the outlined barriers to infectious disease screening. Indeed, there are recent examples showing the effectiveness of patient or peer champions/support in engaging hard-to-reach groups in ID screening programmes. For example, Surey et al. (2019) found that working with peer support workers resulted in a high level of patient engagement, over half of those screened were found to have chronic hepatitis C and of those 38.6% of patients had a favourable treatment outcomes compared to previous standard of care (5%). Similarly, Shaw et al. (2012) found that primary care ‘change champions’ led to a sustained improvement in health care in their case diabetes care pathways in local practice populations, (Shaw et al., 2012) while MackLellan and Stagg's systematic review of the experiences of peer support workers concluded that the strength of peer support workers was to be able to actively engage with marginalised individuals (including migrants) and through this engagement improve patient health outcomes through increase for example in screening initiatives or medication adherence (Maclellan et al., 2015). However, there is a paucity of this approach for ID screening in primary care and requires further empirical research.

Embedding the Health Catch UP! tool into primary care represents a streamlined mechanism to implement multi-disease screening recommended in national and international guidelines on migrant health. Such approaches have proved useful in screening of ID in migrants in primary care (Pareek et al., 2019; Eborall et al., 2020; Sequeira-Aymar et al., 2022; Gonçalves et al., 2022). Studies on the interventions IS-MiHealth (Spain) and COMBAT-ID (UK) found a significant increase in diagnostic yield of infections screened for and acceptability of approach to primary care and patients, (Eborall et al., 2020; Sequeira-Aymar et al., 2022; Gonçalves et al., 2022). The Health Catch-UP! tool contributes and adds to this people not pathogens approach by providing an individualised holistic health check incorporating not only infectious diseases, but additionally non-communicable diseases and adult catch-up vaccinations based on current guidance. This study has strengthened the evidence base for multi-disease screening in primary care finding that it would be well received and utilised by primary health care professionals and outlining barriers and facilitators to implementation. However, there is little known about the effectiveness of the tool on the intermediate clinical outcomes (i.e., whether patients complete the rest of the screening pathway for diagnosis and care) or on the final clinical outcomes (around mortality, morbidity or quality of life) and the cost-effectiveness of it. Further research must investigate the implementation, effectiveness and cost-effectiveness of this intervention with the addition of a community champion, although we acknowledge the challenge in following up migrant populations (Noori et al., 2021; Pareek et al., 2019).

### Strengths and limitations

This study makes an important contribution to the iterative development of ID screening interventions for migrant populations in primary care. Our findings are strengthened by the breath of primary health care professionals interviewed through use of two phases. These phases incorporated the perspectives of both clinical and non-clinical health care staff. Non-clinical staff are often overlooked in research but their views, and engagement are essential to the successful implementation of any primary care intervention and enhance the study's validity. The inclusion of both those who had extensive and no experience in the field of migrant health, and participants working in both migrant sparse and migrant dense rural and urban environments gave our study a breadth of feedback and contributes to the generalizability of our findings.

A key limitation to our study is that those willing to participate, even if they had no specific migrant health experience, are likely to be more interested and therefore informed in this area and their views may not necessarily be representative of the wider UK primary care community. However, we took care to recruit from a large geographical area and our data show that over 50% of participants reported no experience in migrant health. We also recognise that the patient barriers outlined by our study were reported by health care professionals not migrant patients themselves and can only therefore be taken as perceived barriers: inclusion of patient interviews would have further enhanced the validity of this study.

## Conclusions

The recent trends in migration to Europe and current inconsistency in delivery of evidence-based infectious disease screening of migrant groups in primary care creates a major health inequity for this marginalised group with real public health implications This paper has shown that multi-infection screening via embedding digital tools such as Health Catch-UP! in primary health care electronic care records to stratify screening is theoretically acceptable to primary care staff. This approach is promising to engage migrants in screening programmes; however, further studies are essential to ensure ‘real-world’ effectiveness. Such approaches could help to standardise ID screening, improve health outcomes for a marginalised group of the population and reduce health inequalities represented by delays in diagnosis. Any innovation in this area will need to be part of an intervention package co-developed with people with lived experience of migration alongside primary health care professionals to provide a robust support programme including training, support materials and specialist oversight to result in a sustainable public health intervention. Recommendations based on this study and the wider literature have been outlined in box 1 below.

**BOX 1. Recommendations for policy, practice, and research.**

POLICY

• Simplify and standardise primary care specific ID screening and catch-up vaccination guidelines with emphasis on multi-disease screening to aid clinical decision-making with input of PHCP and migrants.
• Introduce primary care ID screening and catch-up vaccination targets with financial incentives, these could focus on localities known to have large migrant populations
• Develop integrated approach to working between primary and secondary care to share responsibility for ID screening and simplify the care pathway for positive results.

PRACTICE

• Identify primary care ID champions to lead screening and vaccination initiatives and motivate staff.
• Increase the use of local patient/peer champions (as seen with BBVs) from GP practice migrant communities to better understand needs, address health concerns and share information to change perceptions around ID screening/vaccination.
• Increase use and development of available culturally competent ID/vaccination patient support materials.

RESEARCH

• Explore use of innovative digital clinical decision support tools such as Health Catch UP! at scale as a way of normalising ID screening/vaccination for at risk populations.
• Explore novel ways to deliver ID screening/vaccination e.g., through community-based interventions or primary care locality hubs

ALL AREAS

Ensure engagement and involvement of migrant community leaders and members is at the forefront of research and policy decisions.

Alt-text: Unlabelled box

## Ethics approval and consent to participate

Ethics was granted by St George's, University of London Research Ethics Committee (2020.00630) and the Health Research Authority (REC 20/HRA/1674). Participant information sheets were circulated, and signed informed consent was acquired prior to telephone interview. Participants consented to audio-recorded interviews. Participants gave informed consent to participate in the study before taking part.

## Consent for publication

Not applicable

## Availability of data and materials

Data are available on reasonable request

## Funding

This study was funded by the NIHR (NIHR300072) and the Academy of Medical Sciences. JC is funded by the National Institute for Health Research (NIHR in-practice-fellowship NIHR 300290 and Research England) FK is supported by a Health Education England/NIHR Academic Clinical Fellowship. AD is funded by the Medical Research Council (MR/N013638/1). SH is funded by the NIHR (NIHR300072; NIHR134801), the 10.13039/501100000691Academy of Medical Sciences (SBF005I1), La Caixa Foundation (LCF/PR/SP21/52930003), Research England, MRC, and WHO. AFC is funded by the NIHR (NIHR Advanced Fellowship NIHR300072) and the 10.13039/501100000691Academy of Medical Sciences (SBF005\1111). All other authors declare no competing interests. The views expressed are those of the authors and not necessarily those of the NHS, the NIHR, or the Department of Health and Social Care.

## Authors' contributions

JC and SH had original research idea and designed the study, JC applied for ethics and ran the study with SH. FK, AD and AC conducted interviews. JC and FK analysed the data with input from whole team. JC wrote initial manuscript with input from FK and SH. SHE, RH, PM, FS, YC, DZ, FW, ICM, TN, AM, ARM and SH contributed to revisions.

## Declaration of Competing Interest

The authors declare that they have no known competing financial interests or personal relationships that could have appeared to influence the work reported in this paper.

## Figures and Tables

**Table:** 

Infectious Disease	Current approach	Illustrative Quotes
**HIV**	Practice based risk stratification (high risk areas)	**GP1:** We will always be offering the patients who are from certain high-risk areas around the world, particularly HIV screening. We would do that if, of course, we've followed the consent and gained consent of those patients. That's predominantly the only other infectious disease I can think of that we would actively do.
	Universal screening at Registration phase for new patients	**GP 16:** We offer all new patients, regardless of where they come from, HIV screening,
	Symptomatic testing by GPs	**GP 2:** any migrants with any sort of unusual presentation I suppose or apparent presentation. And then more broadly anyone with any unusual rash, nonspecific illness, repeat and prolonged or recurrent fever.
**Tuberculosis**	Latent TB screening pathway for migrants arriving in the past 5 years	**GP 4:** If they come from certain countries we offer latent tuberculosis screening, if they've come within the last five years
	Symptomatic	**GP 23:** Only if someone presented with clinical symptoms which are suggestive. Yes, therefore we would but, otherwise, no. So if someone presented with restricted breath and a cough, then absolutely that would be part of it.
	Not done / migrant is referred to secondary care	**PN 15:** Not TB, that's done separately, but's not usually done in primary care, it's done in secondary care that they're directed to
**Hepatitis B and C**	Registration	**PN 7:** We have a policy in our practice to offer HIV to everyone that registers at our practice, not only migrants, but everyone that registers. Well, full screening, really, HIV, hepatitis C and hep B.
	Abnormal liver function tests pathway prompting screening	**GP 9**: They were trying to get on top of screening, so that meant that any raised LFTs [liver function tests] amount was then gone back to… It needed repeating within a certain time, and then that went on to hepatitis C screening.
	Specific practice-based computer prompts from high-risk countries	**HCA 5:** If they are a new patient and they have only just come in and come from a country which is very high in hepatitis, it prompts you straight up that this is what you need to do.
**Sexually transmitted infections**	Young people screened through computer prompts/ patient health checks	**HCA 3:** Young patients [migrants and non-migrants] are kind of… It's in the popup in the corner. So, it's like a reminder to us to ask about sexual health. It prompts saying need to ask if they're sexually active. And if they are, have they had any screening in the past 12 to 18 months? Did they feel it was required?
	Patient request at registration	**PN 3:** Other things we do screen for them is sexual health screening including HIV, and Hepatitis, and we offer them this with discretion, and they tick yes in the box that they would like to have it.
	Symptomatic	**GP 2:** obviously any symptoms or any urinary symptoms I'd often recommend an STI screen.
**Malaria**	Returning traveller	**GP 17:** there's the whole issue of returning travellers and malaria [migrants and non-migrants]. Obviously, if someone's not well and they've just been somewhere, we would screen for malaria.
	Patient request	**GP 24:** I would say we do quite a lot of malaria screening, because a lot of our patients are worried about that, actually. So, that's either led by us or the patient.
**Parasitic infections (and other NTDs including Chagas Disease, Strongyloidiasis, Schistosomiasis, Helminth infections, Leprosy, Yaws etc.)**	Indication from blood test (including eosinophilia or anaemia)	**GP 11**: I mean, there's lots of anaemias and things, worms and so on, that's one thing that always springs to mind, hook worms, etc.
	Risk stratification	**GP 16:** People who have been migrants and go home to visit family and come back, having eaten unhygienic things. One of my patients who insists on eating raw meat every time he goes to Ethiopia and comes back with tapeworms.
	Not done	**GP 2:** I will occasionally think about schistosomiasis and stuff like that. But I don't think that's even on the radar of most doctors. And so, if someone comes in, a migrant with symptoms pertaining to worms or helminth or anything else, I don't think they would even consider it necessarily.

**Table:** 

Level	Solution	Illustrative Quote
Patient Level	Patient engagement using trusted sources from within the community patient-led groups and outreach	**PN13:** we were able to get volunteers from within the Mandarin and Cantonese-speaking communities. There was always a translator. There was always some leaflets and information as well in different languages. So, I think that helps so much. **ADMIN 6:** We have a couple of patient-led groups, and one of the groups that we have is a Somalian women's group, which one of our patients has recently been setting up. And through her work and her outreach in the community, she meets with a lot of Somalian women and talks to them about health matters and community matters.
	Foster greater patient understanding of multi-disease approach, benefits, at first engagement	**GP1**: They have to understand the relevance of it from the outset. Chasing them up later on, is a real challenge. So, it's doing it at the start, and saying… We're doing this as a proactive health check, health screen, to try and look for you, and look for these infectious diseases, is a great opportunity right at the… It's a golden opportunity, right at the beginning of their journey into the NHS system.
	Explore the role of using patient champions to support multi-disease screening and preventative healthcare in migrant patients	**GP5:** And then the HIV screening… The HIV Champions has been useful for GPs, definitely.
Staff Level	Identify an infectious disease champion at practice to oversee training, delivering of services and updating of clinical pathways according to changing guidelines, leading liaison with secondary care	**GP 9**: [with regards viral hepatitis pathway] there was one clinician who led in and oversaw it, rather than being done by lots of different doctors **GP5:** Again, I think relationships do help with the HIV work because we have a GP with a special interest in HIV in the borough who then liaises with the hospital… and the consultant's come around to quite a few practices.
	Quality Improvement Projects specifically aiming to improve understanding and practice around infectious disease screening	**GP19**: lots of local QI projects encouraging awareness of symptoms of IDs, pick up on tests.
	Specific clinics in high-risk areas	**HCA6**: We have a tuberculosis clinic. We have a specialist TB clinic, because we have quite a high prevalence of TB in our borough. And we have a hepatitis clinic
	Feedback to team of positive results and the impact of these	PN14: he'd do a little conference and he just showed how, through our service and their service combined, how the TB cases had dropped in Newham, like huge percentages.
	Engagement of whole primary care team and practice-wide protocol	**GP9:** Well having a practice protocol in place, so having the system, and not just relying on one person, but making sure the team is aware of what to do in the process, which is… Obviously every single one has been looked at and screened and then you have an algorithm-to-follow practice and it's followed up. If it's abnormal, you need to talk to a clinician about having well-trained personnel who follow the protocol properly. That's really important.
System Level	Patient-facing campaigns to improve population understanding of infectious disease screening	**PN9:** because you know on the back of buses, I can see point of care HIV; you can get it done. It's very normalised. You can go down to the local library and get it done. I'd like it to be more of a national campaign that this is available. TB is on the rise, multi drug-resistant TB is frightening. It's exploding in London, and we've got a lot of it out here in Watford as well. Perhaps if there was loads more awareness, we could raise it on the public side but also from the clinicians as well
	Specialist support such as upskilled primary care staff or secondary care outreach clinics	**PN13:** There was a partnership between the clinic I was working in and three specific GP practices who ran this clinic. So, they would identify the particular patients that they needed and then we would go there, and we would just do Hepatitis B screening. **PN13:** I've seen it mainly in TB and it tends to be TB specialist nurses holding clinics within the community……to improve adherence to actually taking medication and then targeting screening for the families or people in close proximity to those people who have tested positive. **HCA6:** And they have a large Somalian support network there, so they have interpreters…. If we feel that they're not really engaging with their condition. If they're hepatitis positive…. we can send them there, and they help us. They will go in, and there will be a Somalian phlebotomist and doctor, and so they engage with it that way, much easier.
	Collaborations between several GP practices to focus on providing services for a larger population (such as through Primary Care Networks- PCN)	**GP20:** I really do think that the way general practice is moving forward, we do need to think about working at PCN level, and maybe there are clinics that are run specifically for this. [infectious disease screening]
	Use of ring-fenced time through targets or financial incentivisation at local or national level (i.e., a commissioned service)	**ADMIN9:** [to increase screening] the best way to do it is to get the CCGs to add it in as a locally commissioned service, so that the doctors feel that they have time that is ringfenced for this kind of activity.
	Easy screening pathways which provide all information or tests concurrently	**PN12**: [LFT/hepatitis pathway] It's very much a one-stop shop for all the clinicians, and it's all on one page. So, anything that can help bring everything and generate all the information on one page, it would be great
	Easy access to tests and follow-up support for patients testing positive	**GP 9:** So, you've got to get your practice systems right, basically, and ensure that the tests that have been asked for, say, or hepatitis or [unclear], chest x-ray, or whatever, are both requested and then followed up as well
	Computer prompts to support clinical decision making	**GP5**: I think if I've got someone in front of me, I'll think about HIV and TB if they're on my screen, because that helps to prompt **HCA 5**: Normally we say where have you come from and when did you arrive in the country. I don't know what countries are at the highest register in terms of if they have got latent TB or not it's TB based. So, if it's on the computer that's a good thing for us
	Competition between practices to demonstrate success of screening programmes	**GP18:** inter-practice kind of competition element, or the inter-federation competition. Or just feedback of rates, like what we're doing compared to how poorly or well we did last year, or you know, what improvement we've made and what difference that's making to people. That is a good way of encouraging people to keep going with something. **GP16:** We're really keen. We've got the highest level of hepatitis B, hepatitis C, and HIV screening running practice in [the borough]. We're doing all right on that. We screen people at the drop of the hat.

**Table:** 

Level	Benefits	Concerns
**Patient Level**	**Provision of good preventative care** **GP15**: think it's quite easy to justify it being a pretty key part of the primary care we can give to migrants, isn't it? I think waiting for people to present with stuff, particularly passive case-finding … is a really bad idea on a public health basis, not just on an individual health basis.	**Stigma of targeted screening** **PN2:** for people coming in now speaking to them and asking them to do that because they've come from X country, it does sound a bit weird, and it's a possible, uncomfortable conversation.
	**One stop shop for patient** **ADMIN 8:** It sounds like a fantastic fishing net, so to speak **GP 1**: universal, one set of tests and blood tests, or urine, would generally be very effective.	**Risk of DNA** **ADMIN 13:** then there are quite a lot of people that we end up not quite persuading, but we may give more information and we then set up the appointment and they don't come. And that's for screening, clinical screening as well.
	**Focus on vulnerable group** **GP2**: It's very well recognised that migrant health outcomes are much lower in many conditions. So, I think it's definitely worth bringing it to the fore of the agenda in trying to address it	**Fear of sharing data (country of origin)** **GP 17**: To find out where someone is born, especially with the present climate, you have to be very careful how you word it. **PN13:** [patients feel] very much that their provision of health care was very linked to their immigration status
**Staff level**	**Everything in one place** **ADMIN 5**: I think that sounds brilliant. It sounds like it would be really helpful. To have everything there in one place **PN 12:** It's very much a one-stop shop for all the clinicians, and it's all on one page. So, anything that can help … generate all the information on one page, it would be great and I'm sure high in demand	**Pop-up fatigue** **ADMIN 6:** Some [staff] just get pop up fatigue. If you have pop ups that come up when they're not relevant to the person and they're not reliable, then you lose the buy-in ……So it needs to be definitely accurate at picking up the eligible people.
	**Reduce workload** **GP20:** Sounds fantastic. To have something like that, that pops up, which is specific for patients from different countries, which means that you're not trawling through evidence, it's just brilliant. **PN 11:** I think because if it pulls together all the information that's there rather than us having to plough through all the previous records to try and get information, that would be a time-saver to begin with.	**Increase workload** **GP6:** Yes, because although part of me feels that's terrible and we should be doing that already. If you're working with a workforce that's already overly stretched, in order to guarantee the practice is able to allocate time and resources for someone to do something extra, you have to justify it
	**Supports clinical decision making** **HCA 2:** Make us more knowledgeable as well and would be easier to be able to say to them this is what we are offering, and this is why. **PHARM 1:** [Health Catch-Up! Would] make us more knowledgeable … and would be easier to be able to say to them [patients] this is what we are offering, and this is why.	**Lack of confidence in infectious disease/migrant health** **ADMIN 5:** here will be quite a few patients that will need these health checks. So that will definitely be a challenge… Making sure we have all the vaccines in stock. Making sure everybody's trained, so they know exactly what these patients need.
**System Level**	**Systematic approach** **HCA1:** I think that would be a really good idea. If it's something that there's searches and …. can identify the people and build it into our general immunisation recall, and if there are clear popups that make it easier for the nurses to opportunistically, and even doctors or reception to make the appointments when they can, then I think that would be helpful	**Incentivisation/financing needed** **ADMIN 15:** It might be a costly and expensive project, but if there is funding available for the practices to target the key demographics or key populations, then, yes, I think there'll be pretty good uptake on it from the practices’ point of view. **GP 21**: finance really important as takes staff time and this may be a particular problem in practices with more migrant patients.
	**Standardisation of screening/vaccination** **PHARM 1**: I think if there are tools that would address the needs of the migrants a bit more specifically, that would definitely make a difference, and standardising it a bit more and make sure that the clinician doesn't miss anything. Yes, I think that would definitely be more useful	**Increased use of appointments** G**P 20**: if they're coming in for their immunisations, it's another appointment ..[there is a problem with] availability of appointments. If you've got a huge cohort coming in now for additional immunisations your nursing appointments will go, and then you have less appointments for other things…I think doing new patient health-checks is good, because it's at a manageable rate.
	**Increase data – improved care/ equality** **PN 7**: (Regarding coding country of origin as part of template) if you do a search and you have it already on EMIS, then you can just run that search from time to time to see who's been registered from abroad and hasn't had any screening. **GP20:** It also means you could probably do searches, isn't it? You could use that information within the template to look at your current cohort and do some retrospective reviews as well. So, overall, really in favour of that**.**	**Difficulty identifying patients** **ADMIN 12**: Sure, the first barrier you'd come across is, how would you identify these patients once they register and what read coding, we're putting in place that will allow you to bring those people up
